# Distinguishing Molecular Properties of OAT, OATP, and MRP Drug Substrates by Machine Learning

**DOI:** 10.3390/pharmaceutics16050592

**Published:** 2024-04-26

**Authors:** Anisha K. Nigam, Jeremiah D. Momper, Anupam Anand Ojha, Sanjay K. Nigam

**Affiliations:** 1 Skaggs School of Pharmacy and Pharmaceutical Sciences, University of California, San Diego, CA 92093, USA; annigam@health.ucsd.edu; 2 Department of Chemistry and Biochemistry, University of California, San Diego, CA 92093, USA; aaojha@ucsd.edu; 3 Departments of Pediatrics and Medicine (Nephrology), University of California, San Diego, CA 92093, USA; snigam@health.ucsd.edu

**Keywords:** drug transport, machine learning, AI, organ crosstalk, gut microbiome, proximal tubule, hepatocyte, SLC22A6, SLC22A8, SLCO1B1, SLCO1B3, ABCC2, ABCC4

## Abstract

The movement of organic anionic drugs across cell membranes is partly governed by interactions with SLC and ABC transporters in the intestine, liver, kidney, blood–brain barrier, placenta, breast, and other tissues. Major transporters involved include organic anion transporters (OATs, SLC22 family), organic anion transporting polypeptides (OATPs, SLCO family), and multidrug resistance proteins (MRPs, ABCC family). However, the sets of molecular properties of drugs that are necessary for interactions with OATs (OAT1, OAT3) vs. OATPs (OATP1B1, OATP1B3) vs. MRPs (MRP2, MRP4) are not well-understood. Defining these molecular properties is necessary for a better understanding of drug and metabolite handling across the gut–liver–kidney axis, gut–brain axis, and other multi-organ axes. It is also useful for tissue targeting of small molecule drugs and predicting drug–drug interactions and drug–metabolite interactions. Here, we curated a database of drugs shown to interact with these transporters in vitro and used chemoinformatic approaches to describe their molecular properties. We then sought to define sets of molecular properties that distinguish drugs interacting with OATs, OATPs, and MRPs in binary classifications using machine learning and artificial intelligence approaches. We identified sets of key molecular properties (e.g., rotatable bond count, lipophilicity, number of ringed structures) for classifying OATs vs. MRPs and OATs vs. OATPs. However, sets of molecular properties differentiating OATP vs. MRP substrates were less evident, as drugs interacting with MRP2 and MRP4 do not form a tight group owing to differing hydrophobicity and molecular complexity for interactions with the two transporters. If the results also hold for endogenous metabolites, they may deepen our knowledge of organ crosstalk, as described in the Remote Sensing and Signaling Theory. The results also provide a molecular basis for understanding how small organic molecules differentially interact with OATs, OATPs, and MRPs.

## 1. Introduction

Many common drugs are organic anions [1]. Along several organ axes—such as the gut–liver–kidney axis—a group of multi-specific organic anion transporters from the SLC and ABC families appear to be responsible for the movement of small molecule drugs (roughly 100–1000 Da), toxins, metabolites, signaling molecules, natural products, gut microbiome products, antioxidants, and vitamins into and out of organs [2,3,4,5]. Understanding the molecular determinants of substrate affinity for these transporters is a key aspect of modern pharmacokinetics, particularly as it pertains to the absorption, distribution, metabolism, and excretion (ADME) of small organic anion drugs [6].

Among multi-specific uptake transporters, the OATs (especially OAT1 or SLC22A6 and OAT3 or SLC22A8) are key to the influx of diverse small organic anion drugs into the proximal tubules of the kidney [7]. For the liver, the main multi-specific uptake transporters are from the OATP (SLCO) family (especially OATP1B1 or SLCO1B1 and OATP1B3 or SLCO1B3) [4]. Among the most important multi-specific ABC transporters involved in efflux (extrusion) of organic anions across the gut, liver, and kidney are members of the MRP family, including MRP2 (ABCC2) and MRP4 (ABCC4) [8]. Antiviral drugs, statins, NSAIDs, and chemotherapeutic drugs can interact with two or more of these six transporters during the passage through organs and body fluid compartments. Defining the substrate specificity of these multi-specific transporters (in the gut, liver, kidney, and elsewhere) is essential to a deeper understanding of how they help coordinate the movement of small organic molecules in the body [9,10,11,12].

The ADME of organic anion drugs along the gut–liver–kidney axis, and inter-organ crosstalk via metabolites and signaling molecules—as, for example, described by the Remote Sensing and Signaling Theory [7,13]—depends to a substantial extent on the ability of this set of six multi-specific transporters to communicate across organs and between tissues and body compartments (e.g., feces, bile, urine). Other multi-specific, oligo-specific, and mono-specific transporters are also involved, but we focus here only on this set of OATs, OATPs, and MRPs, the coordinated functioning of which is clearly necessary for the vectorial transport of organic anions between, for example, the blood and bile and between the blood and urine, as well as between organs.

The polarized localization of these SLC transporters (generally basolateral, uptake) and ABC transporters (generally apical, efflux) is necessary for the coordinated vectorial transport of small molecule drugs and other small molecules across epithelial cells of the gut, liver, and kidney as well as endothelial cells of the brain capillary endothelium (blood–brain barrier) [8]. For example, in the proximal tubule cells of the kidney, OAT1 (SLC22A6) and OAT3 (SLC22A8) are mainly responsible for the basolateral uptake of numerous organic anion drugs (e.g., NSAIDs, antivirals, antibiotics) from the blood into the cell, whereas MRP2 (ABCC2) and MRP4 (ABCC4) are mainly responsible for the efflux of these drugs from the cell into the urine [7].

These SLC transporters (OATs, OATPs) and ABC transporters (MRPs) have many organic anion drug substrates (e.g., methotrexate, statins) in common as well as relatively unique substrates (e.g., tenofovir), the latter type of substrate perhaps being transported mainly by a subset of transporters (e.g., OATs and MRPs but not OATPs). Characterization of the molecular features dictating substrate selectivity for these sets of transporters may facilitate the development of better predictive pharmacokinetic models and an improved understanding of drug–drug interactions or drug–metabolite interactions occurring at the transporter level [14,15].

**Figure 1:**
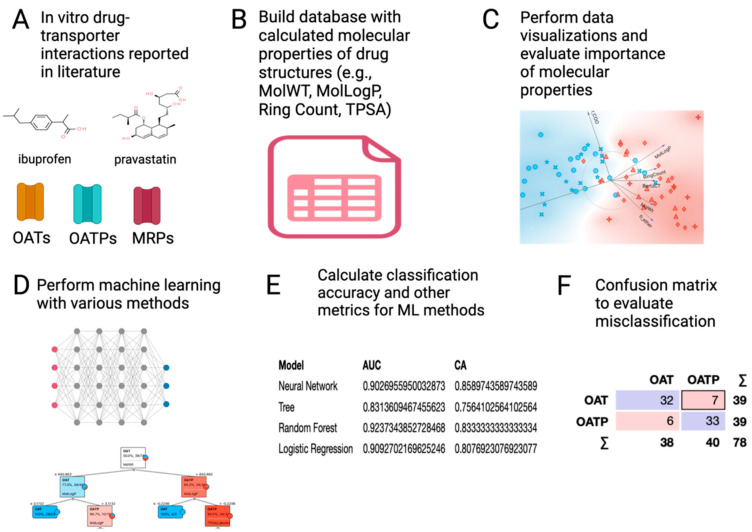
Schematized workflow for identification of molecular properties for binary classification. (**A**) Based on in vitro transport data in the literature, a curated database of drugs interacting with OATs (OAT1, OAT3), OATPs (OATP1B1, OATP1B3), and MRPs (MRP2, MRP4) was created as described in the text. (**B**) Cheminformatics methods (using RDKIT2D) were applied to identify molecular properties (features) of the drugs (instances) and added to the database. (**C**) Data visualization and statistical analysis in Orange, Python libraries, and R (ggplot2) were used to logically narrow down to sets of molecular properties used in machine learning approaches for binary classifications of either OATs vs. MRPs, OATs vs. OATPs, or OATPs vs. MRPs. Particularly useful for choosing features were information gain and the FreeViz diagram. (**D**) Various machine learning classification methods (e.g., Random Forest, Decision Tree, kNN, Naive Bayes, neural network, support vector machines, logistic regression) were applied using the chosen sets of molecular properties (features). (**E**) Classification accuracy and other metrics were calculated. (**F**) Misclassifications of drugs (instances) were evaluated in a confusion matrix. This figure was generated using Biorender.

To this end (Figure 1), we have applied chemoinformatic approaches and machine learning approaches, combined with a variety of data visualization methods, to analyze substrate selectivity of OATs vs. OATPs, OATs vs. MRPs, and OATPs vs. MRPs. Building on our previous work on uptake transporters [16,17], this study provides new insight into how uptake (SLC) transporters function in coordination with efflux (ABC) transporters to mediate vectorial transport of organic anions across the gut, liver, and kidney as well as organ cross-talk.

## 2. Materials and Methods

### 2.1. Data Curation

As indicated in Supplementary Table S1, data from transport assays were curated from a variety of online databases and publications and molecular properties for each drug were assigned by importing RDKit2D into Python. To arrive at a final dataset with ~150 drugs, initially we analyzed drug interaction data for 6 individual transporters, which have been designated as OAT1, OAT3, OAT Both, OATP1B1, OATP1B3, OATP Both, MRP2, MRP4, and MRP Both. However, it soon became evident that for several of the 9 groups, especially the MRPs, there were not enough data for a reliable classification scheme for each of the multi-specific transporters. Thus, to obtain enough unique drug sets to perform the machine learning analyses, we grouped OAT1 and OAT3 into the OATs, OATP1B1 and OATP1B3 into the OATPs, and MRP2 and MRP4 into the MRPs. Hence, there were three classes: OATs, OATPs, MRPs. Although it was challenging to find drugs that had been tested for all transporters—OAT1, OAT3, OATP1B1, OATP1B3, MRP2, and MRP4—it was mostly possible to find drugs that had been tested for members of OATs, OATPs, and MRPs, or two out of the three families. For example, that made it possible to perform three separate balanced binary classifications of non-overlapping drugs in the context of transporter families: OATs vs. MRPs, OATs vs. OATPs, and OATPs vs. MRPs.

### 2.2. Chemoinformatic Approaches and Selection of Molecular Properties

Molecular properties (features) for drugs (instances) were found by importing RDKit2D into Python. Initially, this gave hundreds of molecular descriptors. These were then narrowed down into a set of ~25 mostly interpretable molecular properties. Depending on the separate classifications (OATs vs. MRPs, OATs vs. OATPs, OATPs vs. MRPs), the sets of molecular properties were further narrowed down to 5–10 based on univariate analyses, correlations, information gain, FreeViz analyses, principal component analysis (PCA), and other methods.

### 2.3. Data Mining and Machine Learning

Orange Data Mining software (version 3.29) was used for visual programming and a workflow-based approach to data mining. The Orange software package is based in Python libraries, including Scikit-Learn, Numpy, and Scipy [16]. Various statistical and visualization approaches, including Principal Component Analysis, FreeViz [16], information gain-based ranking, and supervised machine learning-based binary classifications (i.e., k-means classification, decision trees, random forests, neural networks, logistic regression, and Naive Bayes) were utilized [18,19]. Some confirmatory machine learning analyses were carried out using Python Pandas, SciKit-Learn, and Seaborn. Some visualizations were also performed in the R package ggplot2.

## 3. Results

### 3.1. Curation of a List of Drugs Interacting with OATs, OATPs, and MRPs

From pharmacokinetic and physiological analyses, it is clear that OATs, OATPs, and MRPs handle large numbers of organic anion drugs, often with overlapping substrate specificity. Nevertheless, it is also clear from experimental in vitro transport assays that these transporters—while sharing many substrates—can uniquely, or relatively uniquely, interact with particular drugs depending on their molecular properties [16,17,20,21,22].

Our initial goal was to identify a large set of drugs uniquely interacting with OATs (OAT1, OAT3), OATPs (OATP1, OATP3), and MRPs (MRP2, MRP4). This turned out to be challenging, as it is not common that all six transporters are analyzed for the same drug in a single study, making it difficult to identify data collected under the same conditions to assess whether a drug is uniquely transported by OATs, OATPs, and MRPs.

Nevertheless, we identified many drugs that had a strong affinity for OATs compared to OATPs (and vice versa) or OATs compared to MRPs (or vice versa). For OATPs compared to MRPs (and vice versa), it was challenging to find as many drugs that favored one transporter family or the other. This was in part due to another problem encountered; there appeared to be more published data for drugs interacting with the OATs and OATPs than there were for the MRPs. This seems at least partly due to the inclusion of OAT1, OAT3, OATP1B1, and OATP3 in the original “FDA Seven” group of transporters, presumably leading to a greater focus on these transporters over the past decade or so.

All these factors led us to curate a database for the machine learning and data visualization studies that follow. Although compounds in the database of ~150 small organic anion drugs sometimes interacted apparently uniquely with one family (e.g., OATs), they mainly included those that interact “relatively uniquely” (two of the three families). Thus, while the dataset was not ideal for multiple classification, it did appear suitable for binary classification. We thus focused on binary classification of (1) OATs vs. MRPs, (2) OATs vs. OATPs, and (3) OATPs vs. MRPs. However, because of the factors cited above, we also anticipated that unsupervised techniques and complex data visualizations were likely to be useful in the analyses, particularly with respect to OATPs vs. MRPs, where there were fewer drugs uniquely interacting with each family.

### 3.2. Molecular Properties Useful for Classification of OATs vs. MRPs

In the kidney, OAT1 and OAT3 account for by far the largest fraction of the uptake of small organic anion drugs (particularly albumin-bound) from the blood (basolateral side) into the proximal tubules. It is generally held that MRP2 and MRP4 are the major apical transporters responsible for efflux of the bulk of the same (presumably unmodified) organic anions into the urine.

Thus, current views are that the basolateral OATs and apical MRPs work in series to mediate the trans-tubular transport of organic anions from blood to urine. While this indicates substantial substrate overlap, the fact that MRPs are well-known to be involved in handling of larger, more hydrophobic drugs in the liver—compared with smaller, more hydrophilic small molecules handled by the kidney—suggests that there may also be important differences in molecular properties of drugs interacting with the OATs compared to MRPs.

Thus, it is interesting and important to define the substrate selectivity of OATs (OAT1, OAT3, OAT Both) vs. MRPs (MRP2, MRP4, MRP Both). If the two families could be separated by chemoinformatic-derived “interpretable” molecular properties, this could provide considerable physiological and pharmaceutical insight that may improve understanding of how the kidney, by dint of these four transporters, enables vectoral transport (blood to urine) of a large number of organic anions. It may also lead to new approaches for targeting small molecules to or away from the renal organic anion transport system (OATs and/or MRPs). This could be important in, for example, renal disease.

### 3.3. OATs (OAT1, OAT3) vs. MRPs (MRP2, MRP4)

Beginning with OATs vs. MRPs, we performed a number of univariate analyses of the molecular properties of the OAT and MRP drugs, correlations, and other analyses (e.g., Information Gain, FreeViz) to reduce the hundreds of molecular properties calculated by RDKIT to 5–10 interpretable molecular properties that could be used for machine learning classification. The dataset was balanced with 32 OAT-interacting drugs (e.g., penicillin G, acyclovir, naproxen) and 32 MRP-interacting drugs (e.g., losartan, atorvastatin, doxorubicin) with no overlap among the selected sets.

**Figure 2:**
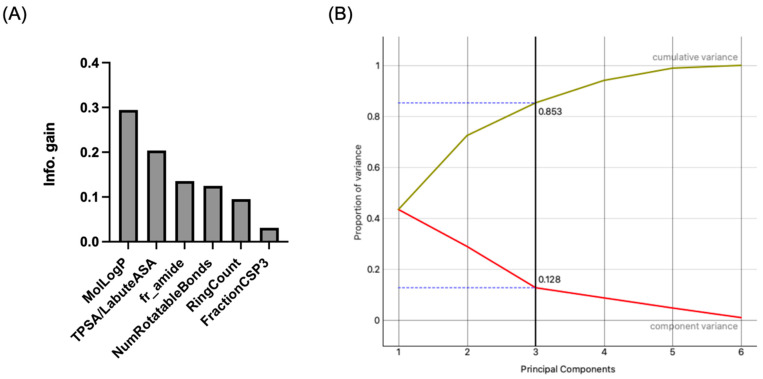
Ranking of molecular properties for binary classification of OAT-interacting and MRP-interacting drugs based on the information gain metric and principal component analysis (PCA). (**A**) Shown is a bar graph of the molecular properties ranked based on information gain. As described in the text, this was one of several methods used to come up with the final set of molecular properties for binary classification. (**B**) Principal component analysis indicates that the initial three principal components explain ~85 percent of the variance.

**Figure 3:**
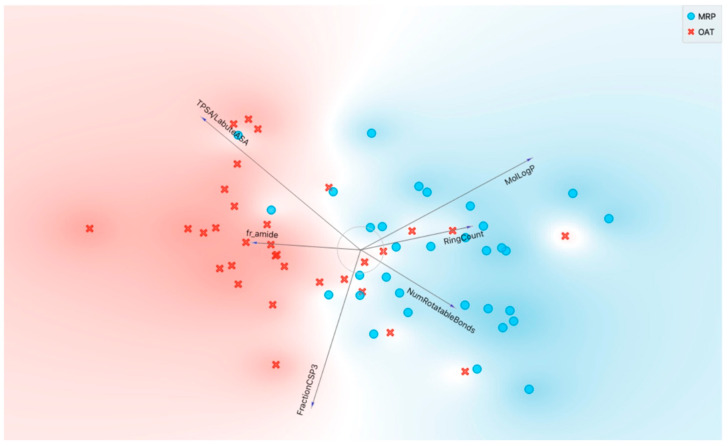
FreeViz diagram depicting distinction of OAT-interacting and MRP-interacting drugs based on selected molecular properties. As described in more detail in the text, the FreeViz diagram is a method to analyze the potential impact of sets of features (molecular properties) in determining the interaction of drugs with OATs vs. those interacting with MRP space. The shading of red and blue indicates the general OAT (OAT1 and/or OAT3) vs. MRP (MRP2 and/or MRP4) space. In the representation, the red crosses depict the OAT-interacting drugs, and the blue circles depict the MRP-interacting drugs. In a FreeViz diagram, the length of the arrow reflects magnitude of the feature according to the FreeViz optimization algorithm in Orange, and the angle between arrows is indicative of the correlation between the features (molecular properties). It is evident that OAT-interacting drugs have a high TPSA/LabuteASA (~surface charge density), and MRP-interacting drugs have a high MolLogP (~hydrophobicity).

Figure 2A shows the final set of molecular properties chosen (MolLogP, RingCount, NumRotatableBonds, FractionCSP3, TPSA/LabuteASA, fr_amide), in terms of Information Gain in a binary classification of OATs vs. MRPs. Visualizations included sets of molecular properties in a FreeViz diagram, where the length of the vector reflects the importance of the molecular property (feature) and the angle between vectors relates to the correlation between various molecular properties (Figure 3). It should be noted that these different analyses may depend on different mathematical approaches/algorithms, so the relative importance of a molecular property may vary with the analysis. However, taken together, they (along with other analyses not shown) demonstrate the potential value of considering this set of interpretable molecular properties for classification of OATs vs. MRPs.

The FreeViz diagram (Figure 3) is particularly helpful for understanding how the OAT-interacting drugs separate from the MRP-interacting drugs based on sets of features. For example, a high molLogP is associated with MRP-interacting drugs, whereas a high TPSA/LabuteASA (an indication of surface charge density) is associated with OAT-interacting drugs. The MRP-interacting drugs that appear to interface with OAT-interacting drugs tend to be those that interact with MRP4 as opposed to MRP2.

**Figure 4:**
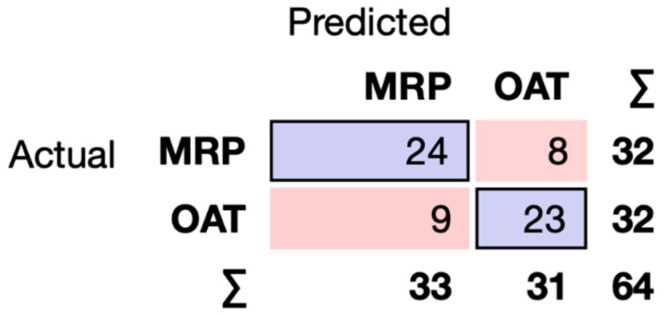
Confusion matrix for random forest binary classifier of OATs vs. MRPs.

By Principal Component Analysis, this set of features accounted for 85 percent of the variance (three components) (Figure 2B). In the Python-based Orange Data Mining environment, we performed Supervised Machine Learning using the aforementioned set of six molecular properties. We analyzed binary classification (OAT vs. MRP) using Decision Trees, Random Forests, Support Vector Machines, Naive Bayes, Logistic Regression, and a simple Neural Network. Several of these performed well using the “leave one out” method (Table 1), yielding 70–75% classification accuracy (e.g., 73% for Random Forests) and reasonable areas under the curve (AUC 0.763 for Random Forests). By confusion matrix, Random Forests misclassification of OATs and MRPs was about the same (nine and eight, respectively, out of a total of seventeen misclassified instances compared to forty-seven correctly classified instances) (Figure 4).

### 3.4. OATs (OAT1 and OAT3) vs. OATPs (OATP1B1 and OATP1B3)

For completion in the context of this study, it was important to analyze OATs vs. OATPs using drugs from the same dataset and using the same methodology. Thus, although we used a different approach for calculating molecular properties (RDKIT2D as opposed to ICM Chemist Pro) and a somewhat different dataset, the binary classification analysis of OATs vs. OATPs came out similar to prior analyses [16]. That said, it is worth emphasizing that, for a balanced set of 78 drugs (39 OATs and 39 OATPs), six molecular properties (MolLogP, RingCount, NumRotatableBonds, MolWt, TPSA/LabuteASA, fr_COO) were sufficient to obtain classification accuracies 75–85% for a wide range of machine learning methods (83% for Random Forests, 82% for Support Vector Machines) with excellent AUCs (≥0.8) (Table 1).

### 3.5. OATPs (OATP1B1, OATP1B3) vs. MRPs (MRP2, MRP4)

The OATPs and MRPs were analyzed as a balanced set with 32 instances from each class (total 64). From initial visualizations of distributions of one molecular property at a time, it soon became clear that there were few clear separations between OATP-interacting drugs (e.g., erythromycin, bosentan, cyclosporine) and MRP-interacting drugs, and that it was likely that more features (molecular properties) would be necessary to classify the OATPs vs. MRPs than were needed for OATs vs. OATPs and OATs vs. MRPs. Indeed, analyses of features based on Information Gain and the FreeViz diagram suggested that close to 10 molecular properties may be necessary for classification. In the end, we chose molLogP, MolWt, RingCount, NumRotatableBonds, FractionCSP3, TPSA/LabuteASA, fr_ether, fr_aniline, fr_amide. Even these only gave classification accuracies of around 55–62% (62.5% for Random Forests).

**Figure 5:**
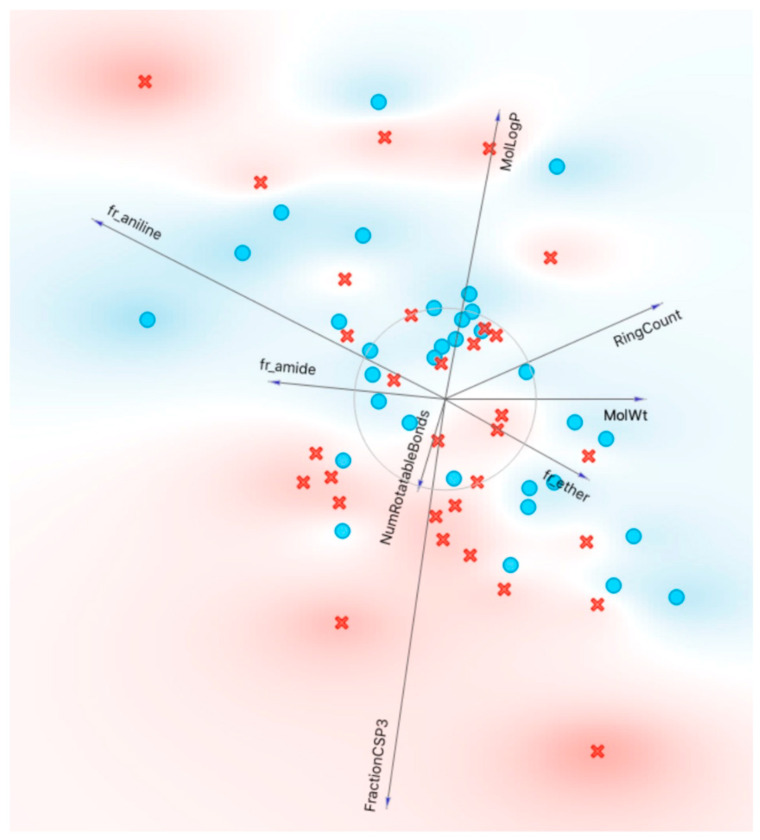
FreeViz diagram depicting distinction of OATP-interacting and MRP-interacting drugs based on selected molecular properties. Red crosses depict the OATP-interacting drugs and blue circles depict the MRP-interacting drugs. When compared to the OAT vs. MRP classification in Figure 3, it is clear that the separation of the red-shaded and blue-shaded areas is less distinct.

**Figure 6:**
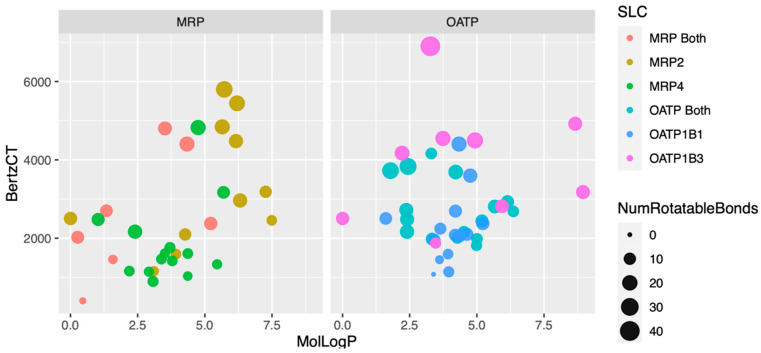
Analysis of OATP- vs. MRP-interacting drugs by MolLogP, BertzCT (measure of molecular complexity), Number of Rotatable Bonds, and Transporter (OATP1B1, OATP1B3, OATP Both, MRP2, MRP4, MRP Both). The graph was created using ggplot2 in R. As with the FreeViz diagram, it becomes clear that MRP2 and MRP4 distribute differently, with MRP2 having a higher molLogP, BertzCT, and NumRotatable Bonds. MRP4-interacting drugs appear more similar to OATP1B1-interacting drugs.

Looking at the FreeViz diagram (Figure 5), it became clear that one of the problems might be the tendency of a large subset of MRP4-interacting drugs to group separately from MRP2 and somewhat more closely with OATP1B1. These tendencies are clearer in the ggplot2 graph made using R (Figure 6).

## 4. Discussion

For small molecule drugs that are organic anions, the multi-specific organic anion transporters of the OAT, OATP, and MRP families may play essential roles in ADME along the gut–liver–kidney axis as well as other axes. A detailed analysis of the molecular basis of substrate selectivity is key to a deeper understanding of pharmacokinetics and for prediction of potential drug–drug and drug–metabolite interactions. Such predictions may have applications both in the drug development setting and clinical practice.

Furthermore, data from metabolomics of knockout mice, as well as metabolic reconstructions, indicate a critical role for these multi-specific transporters in tissue and systemic metabolism as well as organ crosstalk involving organic anion metabolites, many of which are signaling molecules, antioxidants, or participants in key metabolic pathways (e.g., TCA cycle). Organ crosstalk involving metabolites and signaling molecules transported by these multi-specific transporters is a key concept in the Remote Sensing and Signaling Theory [2].

The combination of chemoinformatic approaches, machine learning (ML), and a variety of data visualization techniques allowed us to define important differences in substrate preferences between the OATs, OATPs, and MRPs. Although there are a number of drugs and metabolites handled with differing affinities by at least one OAT, one OATP, and one MRP of the six multi-specific organic anion “drug” transporters studied here, to identify the substrate preferences of each family, we focused as much as possible on unique or relatively unique substrates. Because there was much more in vitro transport data for OATs and OATPs, this was easier to do for the OAT vs. OATP analysis than the analysis involving the MRPs (OATs vs. MRPs, OATPs vs. MRPs), for which there were considerably less transport studies.

The current study has some limitations. To have enough uniquely labeled data to perform the unsupervised and supervised ML studies, we combined OAT1 and OAT3 into the OATs, and likewise, combined OATP1B1 and OATP1B3 into the OATPs and MRP2 and MRP4 into the MRPs. After preliminary analysis of the individual transporters—what have been designated as OAT1, OAT3, OAT Both, OATP1B1, OATP1B3, OATP Both, MRP2, MRP4, and MRP Both—it was clear that for some of the nine groups, especially the MRPs, there were simply not enough instances for reliable classification by a single transporter. Ideally, one would have an independent test set of drugs that interact with OATs and MRPs in order to ascertain the model’s predictive value in a binary classification. However, currently there is a paucity of data on drugs interacting with MRPs, and these data were used to build the model.

Clear differences in substrate preference, consistent with previous analyses [16,17], were found in the OAT vs. OATP analysis, with high classification accuracy and good ROC curves. Similarly, the classification accuracy and other metrics were good for the OAT vs. MRP analysis. However, the classification of the OATPs vs. MRPs was suboptimal using a variety of classification approaches, including Random Forests, Decision Tree, kNN, Naive Bayes, Logistic Regression, and others.

For the OATP vs. MRP analyses, different data visualization tools were particularly useful and revealing. The ML analyses had revealed that OATs preferred smaller, more polar molecules, whereas the OATPs and MRPs preferred larger, more complex, more hydrophobic molecules with a greater number of rings. Thus, it was relatively easy to classify OATs vs. OATPs and OATs vs. MRPs.

But because OATPs and MRPs, grouped as families, both preferred larger, more complex, more hydrophobic molecules with more rings, classification by family membership (based on chemoinformatic analyses of properties of interacting drugs) was not very successful. Perhaps the results would have been better had there been a larger number of MRP drug substrates or had we used more molecular properties (features). However, although our preliminary chemoinformatic analyses gave over a hundred molecular properties, we chose to focus on those that were easily interpretable, such as molecular weight, number of rings, hydrophobicity (logP), and so on. We also aimed to use a ratio of features to instances of 10–20 percent.

Notwithstanding, the data visualizations revealed why OATPs vs. MRPs are difficult to classify. In the visualizations, instances were also labeled as OAT1, OAT3, OAT Both, OATP1B1, OATP1B3, OATP Both, MRP2, MRP4, and MRP Both. From this it became clear that there were major differences between OATP1B1 and OATP1B3, and likewise, between MRP2 and MRP4. Indeed, MRP2 and OATP1B3 tended to group closer that expected to each other, whereas OATP1B1 and MRP4 tended to group closer to each other, and indeed, to OAT1 and/or OAT3 in a number of instances. These are some generalizations, and one should refer to the data visualizations for a more precise picture.

Taken together, the analyses indicate important similarities and differences in substrate selectivity between the OATs, OATPs, and MRPs, and between the individual multi-specific drug transporters themselves. In the context of pharmacokinetics, or organ crosstalk, such as that described in the Remote Sensing and Signaling Theory [2], the analyses may suggest certain “transporter axes” for drugs and metabolites involved in uptake and efflux.

For example, OAT1, OAT3, and OATP1B1—SLC uptake transporters in the kidney and liver—seem better matched to the ABC efflux transporter MRP4. In contrast, OATP1B3 and MRP2, highly expressed, respectively, on the basolateral and apical surface of hepatocytes, seem better matched for vectorial transport in the liver. There is a hint in the visualizations that OAT3 is better matched than OAT1 for interaction with OATP1B3 and MRP2 transported molecules, and thus one might postulate a transporter axis involving OATP1B3 and MRP2 in the liver with OAT3 and MRP2 in the kidney. Such an axis may be important for remote communication of certain larger, more complex, multi-ringed organic anions between the liver and kidney. These ideas need to be tested by experiments in vivo or in multi-organ ex vivo experiments.

While the OAT vs. OATP ML classification results were clear and robust, consistent with previous work [16,17], and the OATs vs. MRPs also had excellent classification metrics, we were unable to obtain clear results for the OATPs vs. MRPs. Indeed, the confusion matrices were quite poor at the level of the family (OATPs vs. MRPs). However, when we examined the four individual transporters (OATP1B1, OATP1B3, MRP2, MRP4) with a number of different types of data visualizations, the difficulty in machine learning classification by family membership became apparent.

For example, in certain visualizations, it was evident that a few drugs corresponding to MRP2 and OATP1B3 bring these two transporters closer together when molecular properties such as MolLogP and complexity (BertzCT) are considered. Interestingly, MRP4 tends to be found closer to OAT1 and OAT3 than MRP2 or OATP1B3, probably because MRP4 appears to have a greater preference for molecules with higher surface charge density (TPSA/LabuteASA). If there had been a considerably larger number of relatively unique MRP2 and MRP4 drugs in the database, it might have been possible to successfully perform ML classification with the individual transporters (OATP1B1, OATP1B3, MRP2, MRP4). Nevertheless, the various data visualizations provide considerable insight into the molecular properties that distinguish drug selectivity by each of these multi-specific drug transporters.

Future work will benefit from larger datasets, particularly for drugs interacting with MRPs. This would allow testing of predictive models on an independent dataset as well as a clearer analysis of the similarities and differences between the six transporters. There is also a great deal of interest in graph neural networks for molecular representation, though it is at yet unclear to what extent these improve upon established methods using molecular descriptors [23].

## Figures and Tables

**Table 1:** Success of different algorithms for binary classification of OAT- vs. MRP-interacting drugs and OAT- vs. OATP-interacting drugs based on classification accuracy and other metrics.

	OAT- versus MRP-Interacting Drugs		OAT- versus OATP-Interacting Drugs	
Model	Area under the ROC Curve	Classification Accuracy	Area under the ROC Curve	Classification Accuracy
SVM	0.823	0.766	0.900	0.821
Lasso Regression	0.863	0.766	0.909	0.808
Random Forest	0.763	0.734	0.924	0.833
Neural Network	0.809	0.734	0.903	0.859
Ridge Regression	0.816	0.719	0.900	0.833
Naive Bayes	0.819	0.688	0.924	0.821
AdaBoost	0.604	0.625	0.796	0.756
Tree	0.631	0.609	0.831	0.756

ROC, receiver operating characteristic. Shown are classification metrics relating to Random Forest, Logistic Regression (Lasso and Ridge Regression), Support Vector Machine, Decision Tree, Naive Bayes, k-Nearest Neighbors, and the “out of the box” Neural Network in Orange. The data shown are using the “leave one out” method. The scores are weighted averages over all classes. Given the relatively good classification accuracies, the misclassifications in the confusion matrices were generally low to modest.

## Data Availability

Data are provided in Supplementary Materials.

## Supplementary Materials

The following supporting information can be downloaded at: https://www.mdpi.com/article/10.3390/pharmaceutics16050592/s1, Supplementary Table S1. Dataset of OAT-, OATP- and MRP-interacting drugs including molecular properties.
